# Paraspinal muscles in individuals undergoing surgery for lumbar spine pathology lack a myogenic response to an acute bout of resistance exercise

**DOI:** 10.1002/jsp2.1291

**Published:** 2023-10-30

**Authors:** Bahar Shahidi, Bradley Anderson, Angel Ordaz, David B. Berry, Severin Ruoss, Vinko Zlomislic, R. Todd Allen, Steven R. Garfin, Mazda Farshad, Simon Schenk, Samuel R. Ward

**Affiliations:** ^1^ UC San Diego Department of Orthopaedic Surgery La Jolla California USA; ^2^ UC San Diego Department of Radiology La Jolla California USA; ^3^ Balgrist University Hospital University of Zurich Zürich Switzerland; ^4^ UC San Diego Department of Bioengineering La Jolla California USA

**Keywords:** degeneration, injury, pain

## Abstract

**Background:**

Lumbar spine pathology (LSP) is a common source of low back or leg pain, and paraspinal muscle in these patients demonstrates fatty and fibrotic infiltration, and cellular degeneration that do not reverse with exercise‐based rehabilitation. However, it is unclear of this lack of response is due to insufficient exercise stimulus, or an inability to mount a growth response. The purpose of this study was to compare paraspinal muscle gene expression between individuals with LSP who do and do not undergo an acute bout of resistance exercise.

**Methods:**

Paraspinal muscle biopsies were obtained from 64 individuals with LSP undergoing spinal surgery. Eight participants performed an acute bout of machine‐based lumbar extension resistance exercise preoperatively. Gene expression for 42 genes associated with adipogenic/metabolic, atrophic, fibrogenic, inflammatory, and myogenic pathways was measured, and differential expression between exercised and non‐exercised groups was evaluated for (a) the full cohort, and (b) an age, gender, acuity, and etiology matched sub‐cohort. Principal components analyses were used to identify gene expression clustering across clinical phenotypes.

**Results:**

The exercised cohort demonstrated upregulation of inflammatory gene IL1B, inhibition of extracellular matrix components (increased MMP3&9, decreased TIMP1&3, COL1A1) and metabolic/adipogenic genes (FABP4, PPARD, WNT10B), and downregulation of myogenic (MYOD, ANKRD2B) and atrophic (FOXO3) genes compared to the non‐exercised cohort, with similar patterns in the matched sub‐analysis. There were no clinical phenotypes significantly associated with gene expression profiles.

**Conclusion:**

An acute bout of moderate‐high intensity resistance exercise did not result in upregulation of myogenic genes in individuals with LSP. The response was characterized by mixed metabolic and fibrotic gene expression, upregulation of inflammation, and downregulation of myogenesis.

## Graphical abstract

Individuals with lumbar spine pathology often demonstrate paraspinal muscle degeneration that does not respond to treatment. Here, we found that muscle in these individuals does not mount a myogenic response after an acute bout of hard resistance exercise.

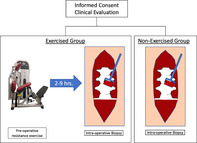

## 1 INTRODUCTION

Lumbar spine pathology (LSP) is often observed in individuals with low back and leg pain, and can cause substantial functional loss and disability.^1^, ^2^, ^3^, ^4^ In individuals with recurrent pain resulting from LSP‐related dysfunction, the paraspinal muscles demonstrate morphological and cellular changes including fatty and fibrotic infiltration,^5^ muscle cell degeneration,^5^, ^6^ infiltration of inflammatory cells, and in some cases, atrophy.^7^ Importantly, individuals with LSP often do not demonstrate normal patterns of hypertrophy or reversal of fatty infiltration in response to rehabilitation.^8^, ^9^, ^10^ This suggests that an impaired adaptive capacity of the muscle could underlie prolonged disability. Muscle recovery potential is influenced by the presence and function of the muscle stem cells (satellite cells)^11^ and well‐defined pro‐myogenic growth pathways.^12^ Muscle hypertrophy is induced by adequate muscle overload, and stereotypic mechanical signaling, gene expression, and protein synthetic responses.^12^, ^13^, ^14^ However, it is unclear if the lack of hypertrophy observed in individuals with spine pain and pathology is the result of insufficient muscle stresses and strains (e.g., low‐intensity exercise regimes due to pain), or an inherent inability of the muscle to mount a hypertrophic response.

Data on muscle responses to exercise vary across species, muscle of interest, age, time of measurement, and exercise intensity.^15^ Stereotypic myogenic genes of interest broadly related to myofibrillar protein synthesis and regeneration include mammalian target of rapamycin (mTOR), myogenic differentiation 1 (MYOD), myogenin, myosin heavy chain‐related genes, and myostatin,^16^, ^17^, ^18^ amongst others. Although acute exercise is assumed to induce adaptive changes that facilitate regeneration, growth and hypertrophy in healthy muscle, the specific genes of interest and their interactions are complex, and remain a focus of continued investigation in the field. In healthy uninjured humans, peak expression of these genes is generally observed 2–12 h after an acute bout of exercise, depending on the gene of interest.^19^ Similarly, activation of pro‐fibrotic, connective tissue elements within skeletal muscle, particularly within the transforming growth factor‐beta  superfamily and Collagens I and III^20^, ^21^ are indicative of muscle fibrosis. Prior investigations have demonstrated a baseline upregulation of fibrotic, atrophic, and adipogenic genes in individuals with LSP,^22^ which are associated with morphological features of muscle degeneration (e.g., fatty infiltration) as observed on magnetic resonance images (MRI).^23^ However, it is unclear if these gene expression signatures influence the adaptive capacity of the muscle in response to an exercise stimulus‐ as would be implemented during rehabilitation. Importantly, although many studies have evaluated the gene expression changes in response to an acute resistance exercise bout in healthy individuals, to our knowledge there is no data evaluating responses in the presence of musculoskeletal disease. Understanding the molecular, cellular, and tissue level responses of muscle to exercise in the presence of pathology will provide an understanding of the disease modifying potential of these common interventions and will be a springboard for identifying more successful intervention approaches. Similarly, identifying defects in the biological responses of muscle to exercise will provide insight into which treatments (or combinations) are likely to resolve structural and functional impairments in diseased muscle. Therefore, the purpose of this study was to compare paraspinal muscle gene expression between individuals undergoing surgery for LSP who do, and do not participate in an acute bout of pre‐operative resistance exercise. Based on fundamental knowledge of muscle responses to exercise, our primary hypothesis was that exercise would induce a promyogenic response as compared to unexercised individuals, and the magnitude of this response would be influenced by the underlying health of the muscle.

## 2 METHODS

### 2.1 Participant characteristics

Participants provided informed consent in concordance with the Declaration of Helsinki and under the approval of the UC San Diego Institutional Review Board (IRB111674). Participants were included if they were undergoing a spinal surgery with a posterior approach, including laminoforaminotomies, laminectomies, discectomies, or fusions (1–2 levels). Patients with diagnosed myopathy, systemic neurological conditions, or traumatic injuries were excluded. All participants had failed attempts at symptom management using conservative strategies (time, physical therapy, activity modification, injections, analgesic medications). Demographic characteristics were collected including age, gender, body mass index (BMI), smoking status (never, past, or current), surgical indication/etiology (Disc Herniation, Stenosis, or Spondylolisthesis), duration of symptoms (months), pre‐operative back and/or leg pain (Numeric Pain Rating Scale [NPRS]), and pre‐operative low‐back pain related disability (Oswestry Disability Index [ODI] Score).

### 2.2 Preoperative diet and exercise bout

**FIGURE 1:**
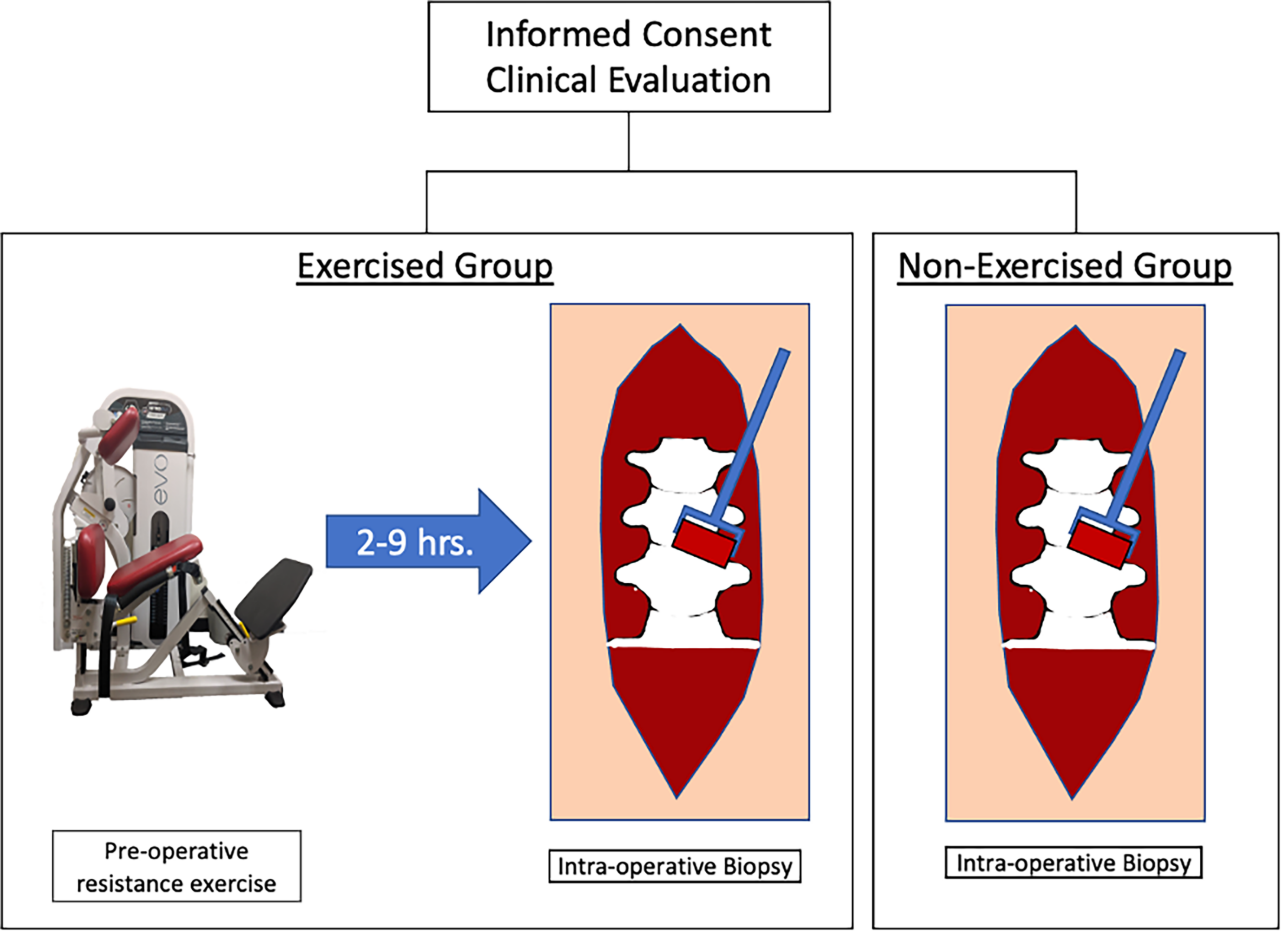
Schematic for study design.

All participants were provided three standardized pre‐surgical carbohydrate drinks composed of 50 g of carbohydrates and 6 g of sugar (Ensure Pre‐Surgery Clear Carbohydrate Drink, Abbott Laboratories Columbus, Ohio). Participants were instructed to drink a shake in the morning and evening the day prior to surgery, and a third shake no later than 4 h prior to scheduled surgery. With the exception of the pre‐surgical shake, participants were instructed to refrain from eating or drinking for at least 8 h prior to exercise. For individuals in the exercise group, an acute pre‐operative machine‐based resistance exercise bout was performed on the day of surgery prior to pre‐operative check in. Participants performed 3 min of self‐paced lumbar extension on a resistance exercise machine (Nautilus Evo) which allows for isolation of the lumbar extensors through stabilization of the pelvis and has been previously shown to elicit measurable hemodynamic and metabolic responses in the paraspinal muscle in individuals with and without back pain.^24^, ^25^ Exercise intensity was normalized to 50% of the participant's body weight, and participants were instructed to pace themselves to achieve a rate of perceived exertion (RPE) of 6–7 out of 10 on the modified Borg Scale of Perceived Exertion. Participants then proceeded to their surgical check in as per standard operative procedures (Figure 1). Exercise resistance intensity (kg), RPE, and time from exercise completion to biopsy acquisition (hours) were documented.

### 2.3 Muscle biopsy and image analysis

Multifidus muscle biopsies were obtained intraoperatively during the posterior approach component of the surgery. Tissue was collected at the level and side of the primary pathology, and was sampled from a standardized anatomical location 1 cm lateral to the spinous process at the spinolaminar border as previously described.^5^ Biopsies were immediately pinned at in‐vivo length and flash frozen using liquid‐nitrogen cooled isopentane, then transported on dry ice for storage at −80°C until processing. To evaluate whole muscle health at the level of the biopsy, pre‐operative T2‐weighted MRIs of the lumbar spine were obtained using a 3 T system (GE MR 750, GE Healthcare, Waukesha, WI, USA) and a spine array coil. Open‐source MRI processing software (Horos) was used to view and analyze axial 2D image slices (4‐mm slice thickness) at the level of the biopsy. Biopsy locations were matched to the MRI slice closest to the inferior vertebral endplate, and level was verified using intraoperative fluoroscopy. From the slice of interest, regions of interest (ROIs) were drawn around the multifidus muscle, and the percentage of fat within the muscle compartment ROI (fat fraction) was calculated using a signal intensity threshold‐based approach as previously described in detail.^26^

### 2.4 RNA isolation and quantitative PCR

Approximately, 25–50 mg of the muscle biopsy was homogenized in a round bottom bead tube (Navy, NextAdvance) with 1 mL of QIAzol (Qiagen). RNeasy spin columns (Qiagen) were used to extract ribonucleic acid (RNA) by following the manufacturer's protocol. Extracted RNA was analyzed for concentration and quality using QIAxpert Analysis (Qiagen). After determining acceptable purity and concentration, complimentary deoxynucleic acid (cDNA) was reverse transcribed using the iScript cDNA Synthesis Kits (BioRad). A standard amount (1 μg) of cDNA per well was loaded in duplicate onto a custom plate (BioRad) to perform quantitative polymerase chain reaction (qPCR) using a BioRad CFX384 Touch qPCR analyzer. The custom plate included a panel of 42 genes associated with regulation of adipogenesis/metabolism, fibrogenesis, inflammation, and skeletal muscle synthesis or degradation (Table 1). Each assay was standardized to an efficiency range of 95%–105% and validated at an annealing temperature of 60°C to allow for gene‐to‐gene comparisons.^27^ Cycle threshold values (Ct values) were determined using a SYBR green fluorophore. Additional on‐plate quality assessment was performed to evaluate genomic DNA contamination and RNA quality.

**TABLE 1:** Genes included on custom qPCR plate.

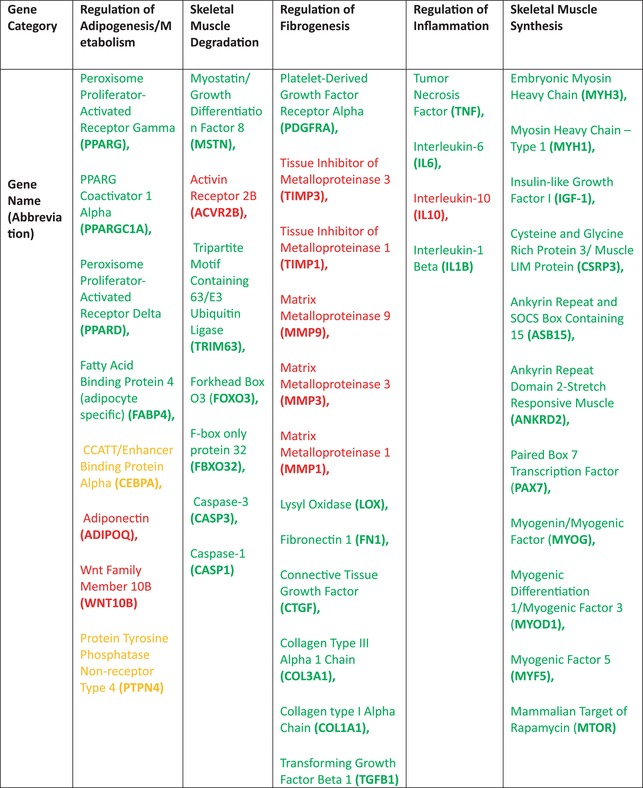

*Note*: Genes that inhibit a given category are indicated in red, those that are facilitatory are indicated in green, and genes that can perform either inhibitory or facilitatory functions indicated in orange.

Abbreviation: qPCR, quantitative polymerase chain reaction.

### 2.5 Statistical Analysis

Raw Ct values were obtained from all samples and read into a qPCR expression set using the R Bioconductor package high‐throughput qPCR (HTqPCR). Ct‐values were quantile normalized to the mean Ct value to obtain gene expression values for each gene, with lower Ct values indicating higher gene expression, and an upper limit of 39 for expression values that were not detected.^22^ Unsupervised hierarchical clustering using Euclidean distances was performed on the normalized Ct values to generate heat maps of gene expression across all patients. Binary logarithm of the fold changes for differential expression values (delta‐delta‐Ct) were calculated with the limmaCtData wrapper in HTqPCR using a moderated *t*‐test between exercised and non‐exercised cohorts.^28^ All raw *p*‐values were adjusted for within‐gene group multiple comparisons using the Benjamini and Hochberg (BH) method.^29^ Significance was set at a BH‐adjusted *p*‐value of *p* < 0.05, and trends were defined as BH‐adjusted *p*‐values of *p* < 0.08. A principal components analysis was performed using the PCAtools function within the R Bioconductor package to evaluate feature clustering across patients and clinical phenotypes associated with feature components. For genes that were differentially expressed between exercised and non‐exercised groups, associations with underlying muscle quality phenotype (amount of fatty infiltration) were evaluated using Pearson correlation coefficients adjusted for multiple comparisons.

Due to the discrepant sample sizes between the exercised (*N* = 8) and unexercised (*N* = 53) samples, a matched sub‐analysis was also performed. Participants in the exercise group were matched one‐to‐one with participants in the non‐exercised group based on age (within 3 years), gender (exact match), etiology (89% match), biopsy level (exact match), and duration of symptoms (acute; <3 months, or chronic; >3 months). Hierarchical clustering and differential expression analyses were repeated on the matched subgroups.

## 3 RESULTS

### 3.1 Participants

Sixty‐four participants consented to intra‐operative muscle biopsies, of whom eight consented to the additional pre‐operative exercise protocol (NCT03442374). Three participants in the non‐exercised group were excluded due to poor RNA quality of their tissue sample, leaving a total of 61 participants (*N* = 53 non‐exercised, *N* = 8 exercised) with complete gene expression data for analysis. Five participants (one exercised, four non‐exercised) did not complete a baseline ODI questionnaire. Participants were, on average, males in their fifth decade of life, with a primary surgical indication of chronic radiculopathy resulting from disc herniation. The majority of biopsies were obtained from the L4 lumbar level, and participants were experiencing moderate pain and disability. There were no significant differences in baseline characteristics between the exercised and non‐exercised cohorts, with the exception of a trend (*p* = 0.06) for a greater proportion of individuals with spondylolisthesis in the exercise group (Table 2).

**TABLE 2:** Patient demographics for exercised and non‐exercised participants.

Mean (SD) or *N* (%)	Non‐exercised (*N* = 53)	Exercised (*N* = 8)	*p*‐value
Age (years)	53.98 (17.06)	58.13 (12.91)	0.51
Gender (*N*, % male)	33 (62.3%)	6 (75.0%)	0.70
Smoking (*N*, %)			1.0
Never	29 (54.7%)	5 (62.5%)	
Past	8 (13.1%)	1 (12.5%)	
Current	16 (30.2%)	2 (25.0%)	
BMI (km/m^2^)	28.28 (5.08)	26.54 (4.25)	0.36
Etiology (*N*, %)			*0.06*
Disc herniation	26 (49.1%)	4 (50.0%)	
Stenosis	17 (32.1%)	0 (0%)	
Spondylolisthesis	10 (18.9%)	4 (50.0%)	
Biopsy level (*N*, %)			0.63
L1	1 (1.9%)	0 (0%)	
L2	0 (0%)	0 (0%)	
L3	15 (28.3%)	1 (12.5%)	
L4	23 (43.4%)	5 (62.5%)	
L5	14 (26.4%)	2 (25.0%)	
Fatty infiltration (%)	39.94 (11.09)	37.31 (14.21)	0.55
Duration of symptoms (months)	24.91 (45.77)	48.00 (45.67)	0.19
Pain (NPRS)	5.64 (2.80)	4.12 (2.80)	0.16
Disability (ODI)^a^	45.06 (21.68)	42.00 (19.87)	0.73

*Note*: Trending *p*‐values are indicated in italics.

Abbreviations: BMI, body mass index; NPRS, Numeric Pain Rating Scale; ODI, Oswestry Disability Index.

^a^ *N* = 56 completed questionnaires.

The participants who underwent the acute pre‐operative exercise bout resisted an average load of 33.7 (7.2) kg over the 3‐min period and reported an average of 6.25 (“hard” exercise) on the 10‐point RPE scale. The average time from the exercise bout to the biopsy was 6.02 (2.78) h, which is within the goal time frame for post‐exercise peak gene expression (Table 3).

**TABLE 3:** Resistance exercise characteristics for participants in the pre‐operative exercise group.

	Exercise resistance (kg)	RPE (pts)	Time to biopsy (h)
Mean (SD)	33.7 (7.2)	6.25 (0.46)	6.02 (2.78)
Range	22.6–43.1	6–7	1.65–9.83

### 3.2 Full‐cohort differential expression

**FIGURE 2:**
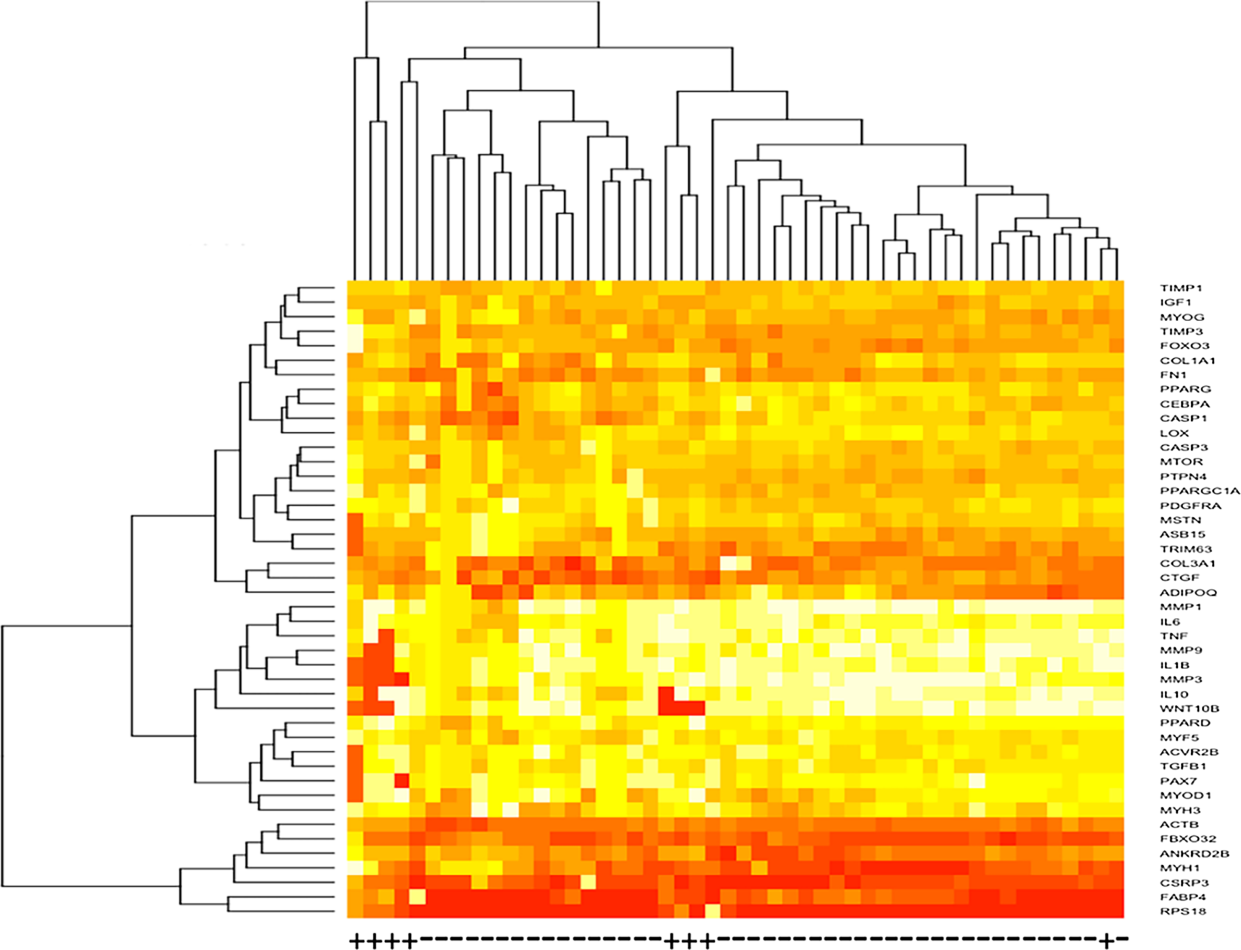
Heatmap for all samples. Unbiased hierarchical clustering of all genes across exercised (+) and non‐exercised (−) samples. Higher gene expression is indicated in red, and lower gene expression is indicated in white.

Hierarchical clustering heatmaps demonstrated that overall, gene expression was low for most genes. Genes that demonstrated expression in the highest quantile included muscle synthesis (i.e., MYH1, CSRP3), atrophic (i.e., FBX032), and regulation of adipogenesis and metabolism (i.e., FABP4) genes. Genes that demonstrated expression in the lowest quantile included inflammatory genes (i.e., IL10, IL1B, IL6, TNF) and inhibitors of extracellular matrix deposition (i.e., MMP1, MMP3, MMP9) (Figure 2).

**FIGURE 3:**
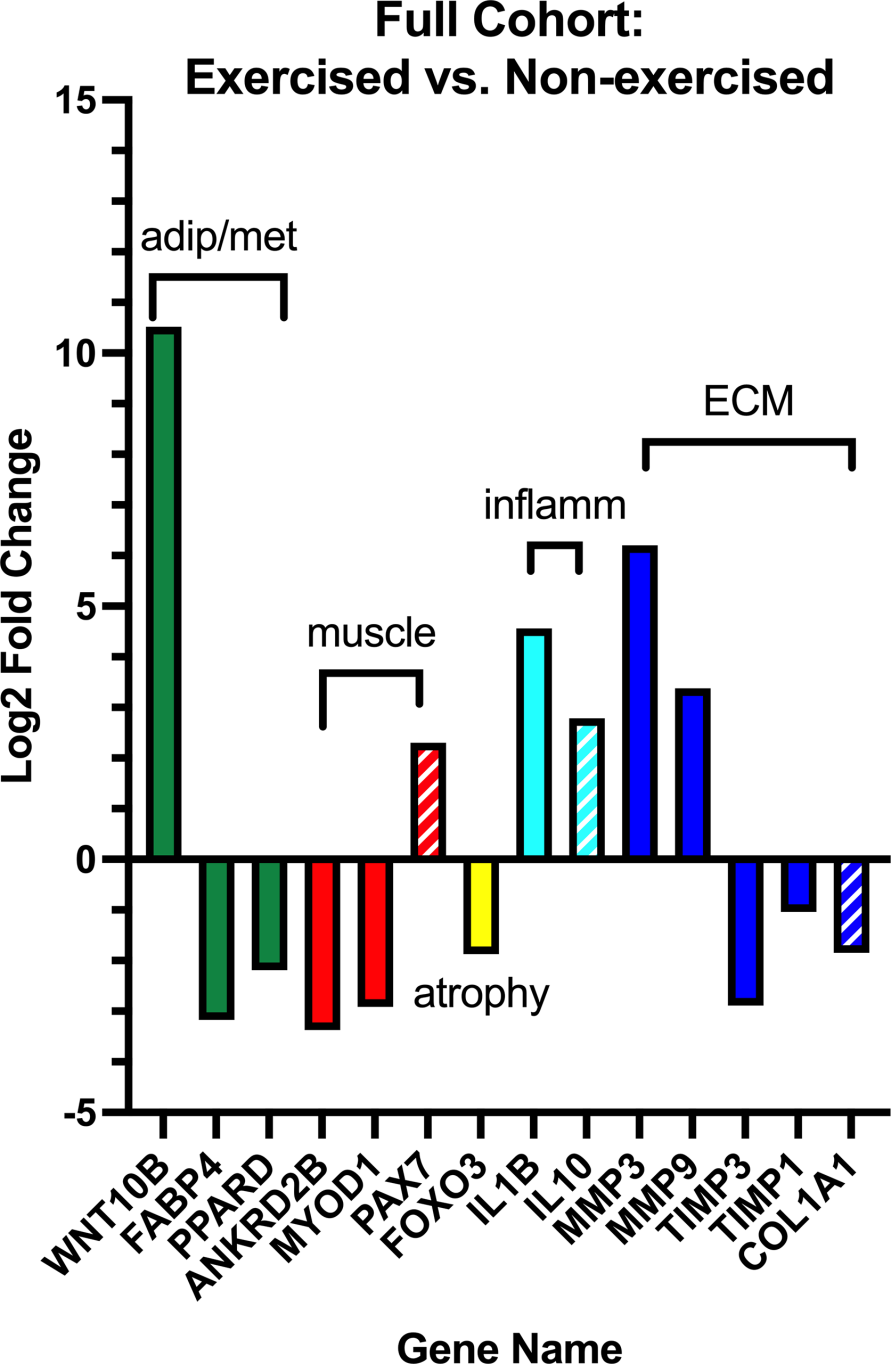
Differential expression of genes between individuals who underwent pre‐operative exercise compared to those who did not. Values are log_2_ fold change, solid bars indicate genes with Benjamini and Hochberg (BH)‐corrected *p*‐values of <0.05, hatched bars indicate genes with BH‐corrected *p*‐values of <0.1.

The exercised cohort demonstrated upregulation of inflammatory genes, mixed expression of lipid metabolism genes, and downregulation of extracellular matrix deposition‐related, muscle synthesis related, and atrophic genes compared to the non‐exercised cohort (Figure 3). Specifically, individuals in the exercised group demonstrated strong upregulation of lipid metabolism gene WNT10B (log_2_FC = 10.5, *p* < 0.0001), and downregulation of FABP4 (log_2_FC = −3.1, *p* = 0.01) and PPARD (log_2_FC = −2.1, *p* = 0.002). In the muscle synthesis pathway, exercise induced downregulation of ANKRD2B (log_2_FC = −3.4, *p* = 0.007) and MYOD1 (log_2_FC = −2.9, *p* = 0.03), with a trend for upregulation of PAX7 (log_2_FC = 2.3, *p* = 0.08). Atrophic gene FOXO3 was downregulated in the exercise group (log_2_FC = −1.9, *p* = 0.04). Inflammatory gene IL1B was significantly upregulated in the exercise group (log_2_FC = 4.6, *p* < 0.0001), and there was a trend for upregulation of anti‐inflammatory IL10 (log_2_FC = 2.8, *p* = 0.07). Finally, extracellular matrix inhibitor genes MMP3 (log_2_FC = 6.2, *p* = < 0.0001), MMP9 (log_2_FC = 3.4, *p* = 0.005), were upregulated and extracellular matrix deposition genes TIMP3 (log_2_FC = −2.9, *p* = 0.002), and TIMP1 (log_2_FC = −1.0, *p* = 0.005) were downregulated with a trend for downregulated COL1A1 (log_2_FC = −1.9, *p* = 0.06) as compared to the non‐exercised group.

### 3.3 Principal components analysis

**FIGURE 4:**
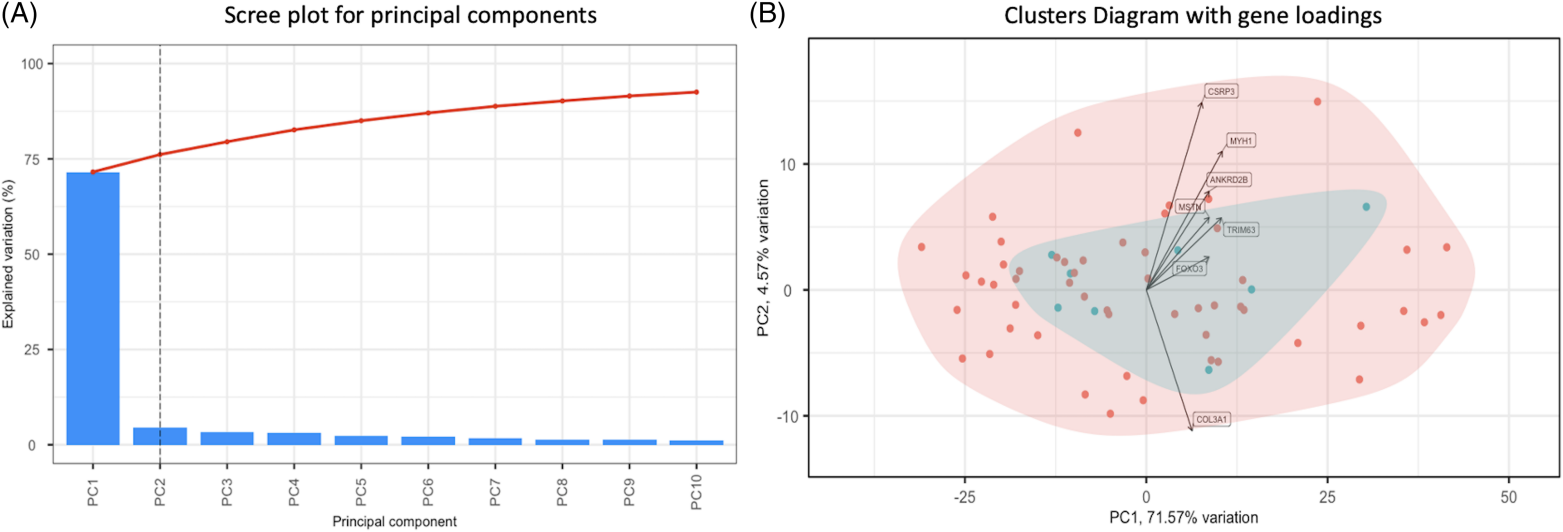
Principal components analysis scree plot (A) and cluster diagram of non‐exercised cohort (red) and exercised group (green), along with gene loadings (B).

**FIGURE 5:**
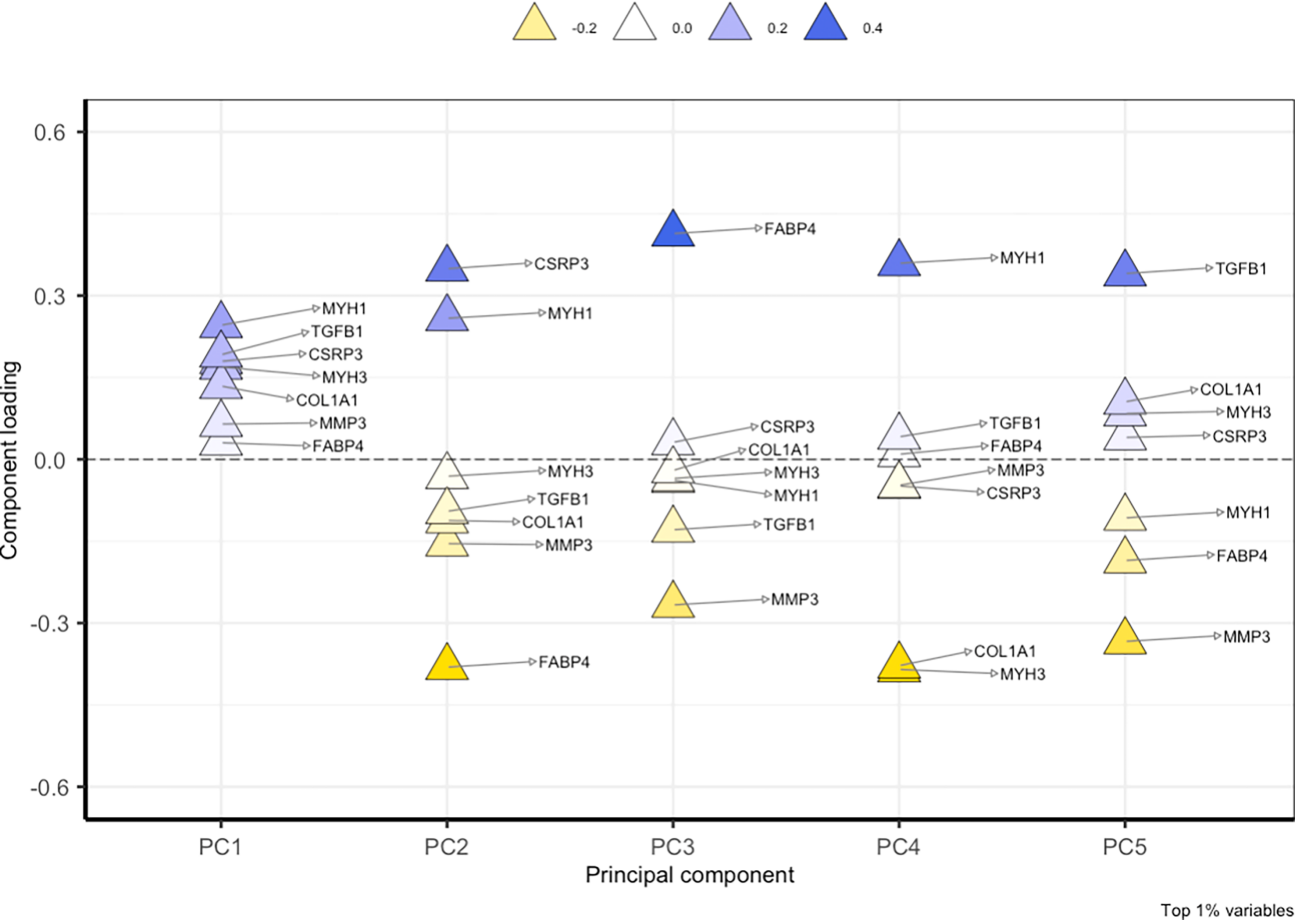
Gene loadings for each principal component up to an 85% variance limit.

The full‐cohort principal components analysis revealed two primary components (PCs); PC1 explaining 71.6% of the variability in the sample, and PC2 explaining 4.6% of the variability (Figure 4A). Variability across the two primary PC's was driven by expression of muscle synthesis‐related (CSRP3, MYH1, ANKRD2B), extracellular matrix deposition (COL3A1), and atrophic (TRIM63, FOXO3, MSTN) genes (Figure 4B), but none of this variability was explained by the presence of exercise. 85% of the variability was explained by the first five PCs, and component loadings for individual genes contributing to variation amongst each cluster can be visualized in Figure 5.

**FIGURE 6:**
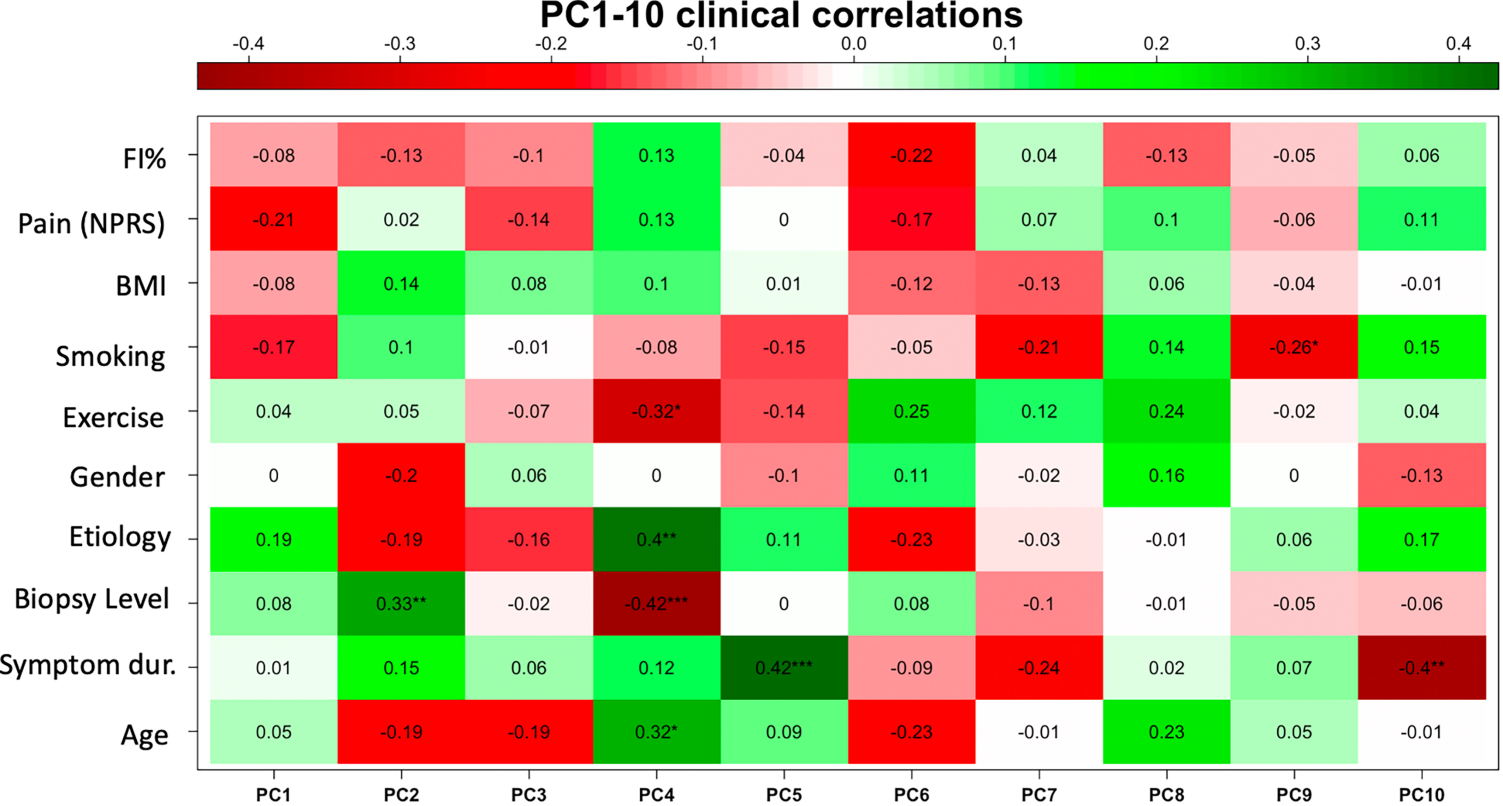
Association of clinical phenotypes with individual principal components.

There were no clinical phenotypes that were significantly associated with the primary principal component (PC1); however, some variability in PC1 was explained by weak associations with pain level (*r* = −0.21), etiology (*r* = 0.19), and smoking status (*r* = −0.17). Biopsy level was significantly associated with PC2 (*r* = 0.33, *p* < 0.01). Exercise status (*r* = −0.32, *p* < 0.05), etiology (*r* = −0.40, *p* < 0.01), and biopsy level (*r* = −0.42, *p* < 0.001) were associated with PC4, and symptom duration (*r* = 0.42, *p* < 0.001) was associated with PC5 (Figure 6). There were no associations between the magnitude of gene expression for the differentially expressed genes in response to exercise and the underlying muscle quality as measured by amount of fatty infiltration (FI%) at the whole muscle level (p > 0.21).

### 3.4 Matched sub‐analysis

There were no significant differences between any of the patient characteristics of the exercised and non‐exercised groups for the sub‐analysis with patients matched for age, gender, etiology, biopsy level, and symptom duration (Table 4). However, the non‐exercised cohort demonstrated pain and disability levels that were clinically (more than the minimal clinically important difference for NPRS^30^ and ODI^31^, ^32^), but not statistically (*p* > 0.12), greater than the exercised cohort.

**TABLE 4:** Patient characteristics for matched samples.

Mean (SD) or *N* (%)	Non‐exercised (*N* = 8)	Exercised (*N* = 8)	*p*‐value
Age (years)	56.00 (9.82)	58.13 (12.91)	0.72
Gender (*N*, % male)	6 (75.0%)	6 (75.0%)	1.0
Smoking (*N*, %)			0.67
Never	3 (37.5%)	5 (62.5%)	
Past	2 (25.0%)	1 (12.5%)	
Current	3 (37.5%)	2 (25.0%)	
BMI (km/m^2^)	28.70 (4.30)	26.54 (4.25)	0.33
Etiology (*N*, %)			0.62
Disc herniation	4 (50.0%)	4 (50.0%)	
Stenosis	1 (12.5%)	0 (0%)	
Spondylolisthesis	3 (37.5%)	4 (50.0%)	
Biopsy level (*N*, %)			1.0
L1	0 (0%)	0 (0%)	
L2	0 (0%)	0 (0%)	
L3	1 (12.5%)	1 (12.5%)	
L4	5 (62.5%)	5 (62.5%)	
L5	2 (25.0%)	2 (25.0%)	
Fatty infiltration (%)	31.80 (9.01)	37.31 (14.21)	0.37
Duration of symptoms (months)	55.63 (105.90)	48.00 (45.67)	0.85
Pain (NPRS)	6.25 (2.25)	4.12 (2.80)	0.12
Disability (ODI)	51.33 (13.00)	42.00 (19.87)	0.17

Abbreviations: BMI, body mass index; NPRS, Numeric Pain Rating Scale; ODI, Oswestry Disability Index.

**FIGURE 7:**
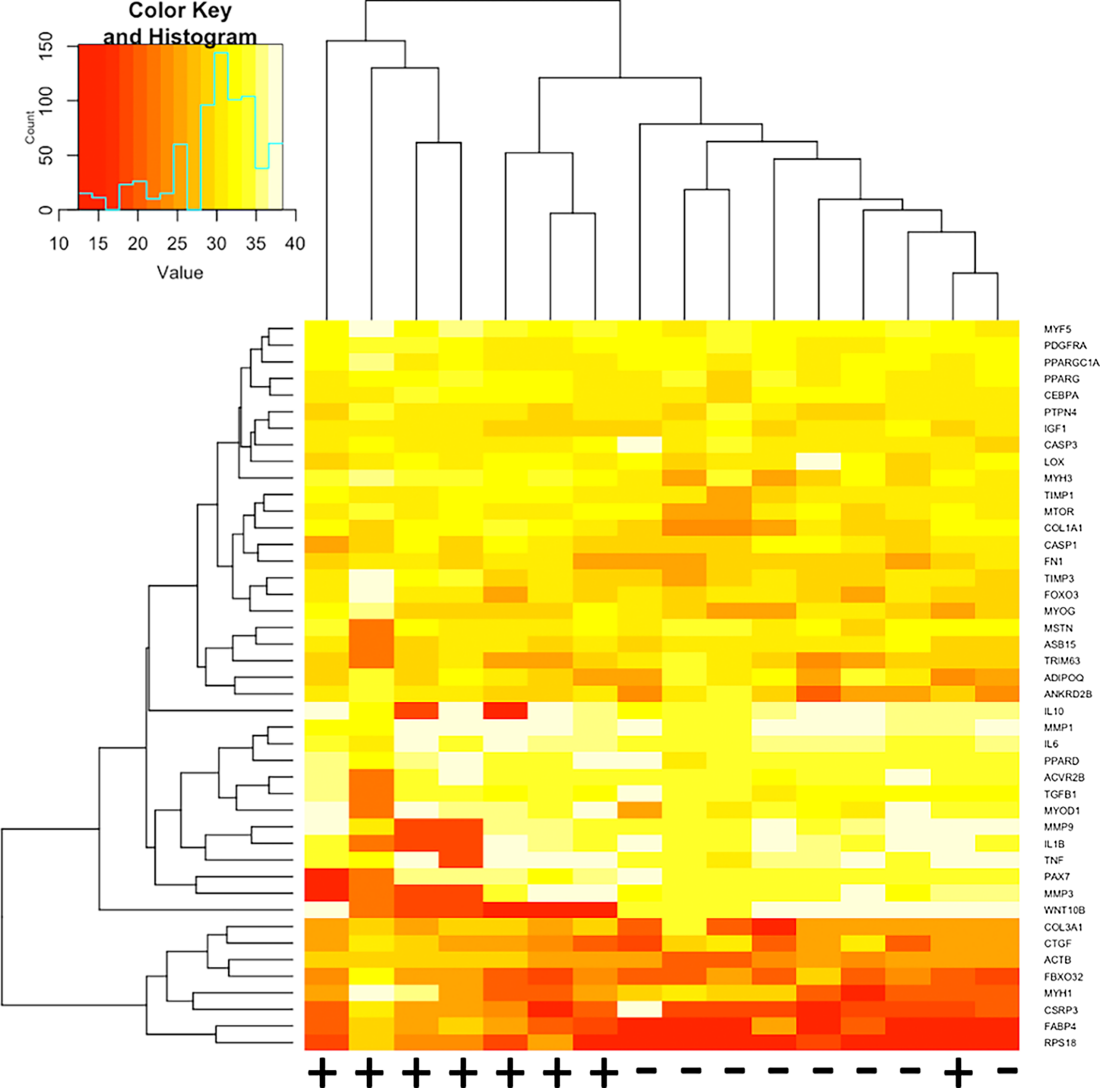
Heatmap for matched samples cohort. Unbiased hierarchical clustering across exercised (+) and non‐exercised (−) samples.

**FIGURE 8:**
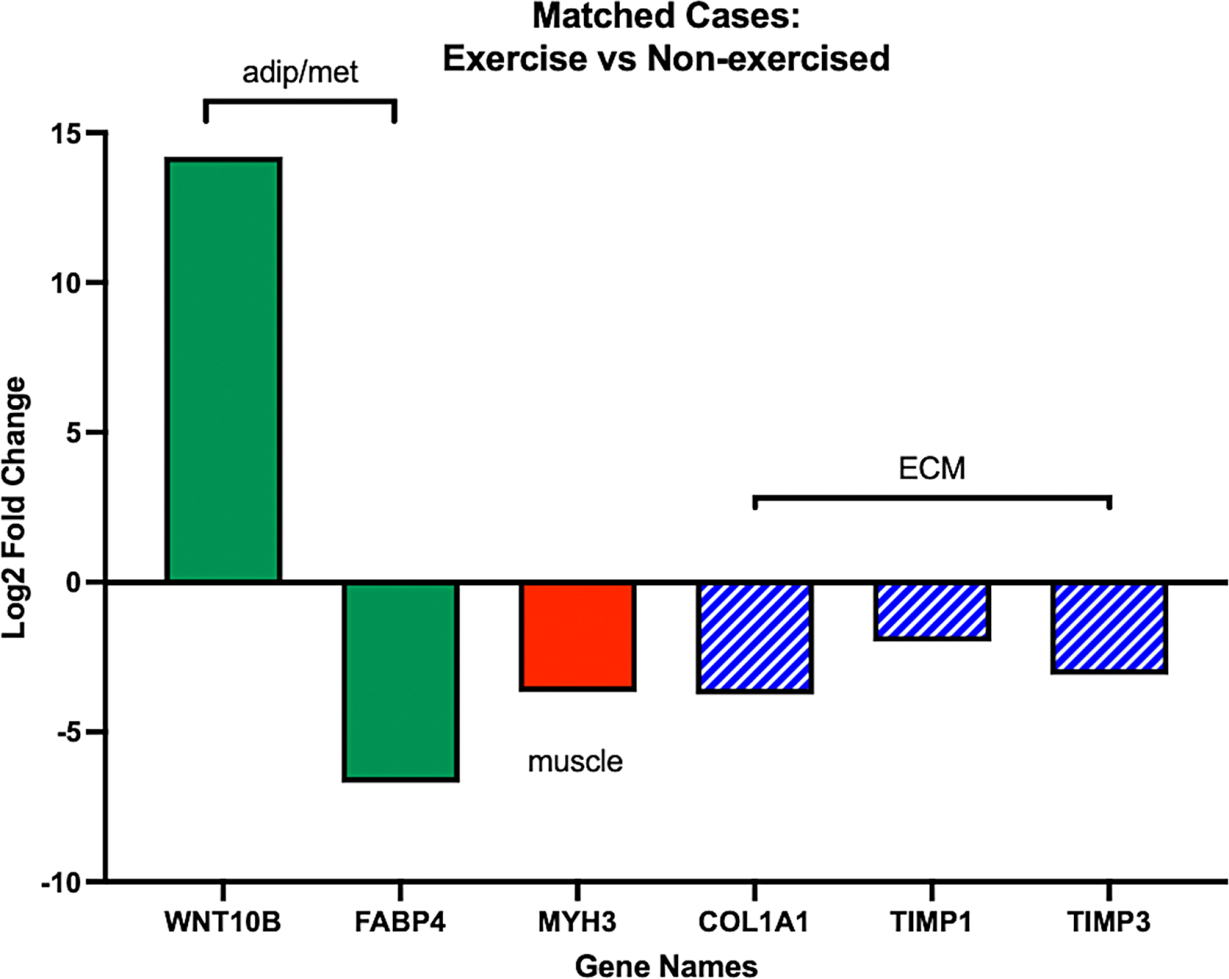
Differential expression of genes significantly different between individuals who underwent pre‐operative exercise compared to those who did not, matched for age, gender, etiology, biopsy level, and symptom duration. Values are log_2_ fold change, solid bars indicate genes with corrected *p*‐values of <0.05, hatched bars indicate genes with corrected *p*‐values of <0.1.

The matched group hierarchical clustering sub‐analysis demonstrated clustering of the exercise group (Figure 7), with differential gene expression profiles across the exercised and non‐exercised groups that were similar to the full‐cohort comparisons. Specifically, there was a strong upregulation of adipogenic gene WNT10B (log_2_FC = 14.17, *p* = 0.004), downregulation of FABP4 (log_2_FC = −6.70, *p* = 0.02), and downregulation of muscle synthesis‐related gene MYH3 (log_2_FC = −3.67, *p* = 0.04). There were trends for downregulation of extracellular matrix deposition genes COL1A1 (log_2_FC = −3.76, *p* = 0.07), TIMP1 (log_2_FC = −1.98, *p* = 0.07), and TIMP3 (log_2_FC = −3.10, *p* = 0.07) (Figure 8).

## 4 DISCUSSION

The purpose of this investigation was to evaluate the gene expression response of paraspinal muscle to acute resistance exercise in individuals undergoing surgery for LSP. Contrary to our hypothesis, we found that exercise did not result in upregulation of myogenic genes in these individuals, and the primary response was characterized by inhibition of extracellular matrix deposition and mixed metabolic gene expression, upregulated inflammatory gene expression, and downregulation of myogenesis. We also observed that the magnitude of gene expression response to exercise was not associated with underlying muscle quality. These data are the first to evaluate the responsiveness of muscle in the presence of musculoskeletal pathology to an acute bout of resistance exercise.

### 4.1 Non‐exercised gene expression in the presence of pathology

Our data demonstrated that most genes evaluated were not highly expressed in this patient population, despite standardization of cDNA amounts and assay conditions across genes and samples. Within our control sample, genes related to muscle synthesis and fat metabolism demonstrated the greatest expression, whereas genes regulating inflammation and extracellular matrix deposition demonstrated lowest expression. However, the same absolute expression levels between genes can have substantially different downstream functional consequences, and caution should be taken when interpreting the biological relevance of these observations in the absence of mechanistic studies and a healthy comparison. Prior literature is sparse related to the patterns of paraspinal muscle gene expression in the presence of pathology. In animal models of disc herniation injury, mixed expression of atrophy (MSTN), metabolism (PPARGC1A), and muscle synthesis (mTOR, IGF1) genes have been observed compared to uninjured sheep.^33^ In addition, expression of inflammatory genes TNF‐alpha and IL1B have been associated with greater fatty and fibrotic changes in the muscle post disc injury.^34^ In humans, individuals with chronic LSP of varying etiologies demonstrate increased fibrogenic gene expression compared to those with acute spine pathology.^22^ In individuals with lumbar kyphosis, expression of PPARGC1A, TNF, and IL6 in the surrounding paraspinal muscles is associated with magnitude of deformity.^35^ Similarly, the magnitude of fibrotic (COL3A1), adipogenic (PPARD), and muscle synthesis‐related (mTOR) gene expression has been found to be associated with paraspinal muscle fatty infiltration and cross‐sectional area.^23^ These data support the concept that metabolic, inflammatory, and extracellular matrix deposition gene expression reflects functional and morphological observations in humans with spine pathology.

### 4.2 Exercise response in muscle

The response of healthy uninjured skeletal muscle to resistance exercise has been described in both animal and human models,^15^, ^36^ across genders,^37^ and over the spectrum of age.^38^ Models of response to an acute bout of resistance exercise demonstrate activation peaking between 2 and 8 h after exercise, although these response times depend on the specific gene of interest and can be shown to maintain elevated activation for up to 48 h after the exercise bout.^19^, ^36^, ^39^ As such, there is large variability in gene expression response over time and across different muscles. Some literature supports the involvement of the PI3K‐AKT/MTOR pathway in animal models of muscle hypertrophy, though the time course of its activation after exercise is not well described and is in some cases conflicting in its relationship with downstream structural changes.^12^, ^16^, ^40^, ^41^ Similarly, other myogenic genes such as Myogenin, MYOD, and MRF4 have been shown to be upregulated at timepoints ranging between 2 and 12 h after acute exercise in both young and old individuals,^19^, ^38^ and a decrease in the expression of MSTN, which can block muscle growth, is also commonly observed.^15^ In parallel, genes involved in extracellular matrix remodeling (e.g., COL3A1, CTGF)^42^ and mitochondrial biogenesis (e.g., PPARG, PPARGC1A) are also activated^14^, ^42^ along with inflammatory genes.^39^ In this study, we did not observe a clear upregulation of genes involved in muscle synthesis. Instead, we observed further induction of metabolic, inflammatory, and extracellular matrix deposition gene programs with downregulation of myogenic genes. The strongest response observed was for the WNT10B gene, which is part of the wingless‐type MMTV integration site family. It is thought to control adipogenic potential in myoblasts and regenerating muscle, inhibits adipogenic differentiation,^43^ and is upregulated in response to exercise for certain types of adipose tissue.^44^ This, along with modulation of the PPARD and FABP4 genes, suggests that the resistance exercise bout may have resulted in activation of lipid metabolism programming. This is in line with the expected metabolic demand associated with acute exercise and is consistent with prior literature.

### 4.3 Exercise response in the presence of LSP

Despite the large amount of literature regarding gene expression responses to exercise, to our knowledge, no investigations have evaluated the response of pathological muscle to acute resistance exercise, or its response in the presence of musculoskeletal pain/pathology. In the absence of such literature, direct comparisons between our results and prior investigations were not possible. However, muscle gene expression in an elderly population is often used as a proxy for these conditions with the rationale that similar phenotypic and functional changes, such as muscle atrophy, fatty infiltration, and strength loss are observed in this population. Indeed, studies in aging populations have demonstrated attenuated gene expression both at rest, and in response to acute resistance exercise when compared to their young counterparts.^45^ For example, Dennis et al. found that in contrast to a young healthy cohort, acute high intensity resistance exercise did not induce significant changes in gene expression in healthy aged participants 72 h after exercise for a panel of 100 genes representing muscle growth and adaptation.^45^ However, they did observe decreased expression of insulin growth factor (IGF1), and matrix metallopeptide (MMP2) in the elderly group at rest. Similarly, another study demonstrated overall reduced transcriptional activity in elderly participants.^46^ In contrast, other studies have demonstrated higher expression of myogenic (MYOD, myogenin) and atrophic (MSTN) genes at rest in elderly women,^38^ and inflammatory and stress response genes 4–24 h after resistance exercise in elderly patients as compared to a healthy young cohort.^46^, ^47^ Overall, studies in healthy aging populations seem to demonstrate some similar gene expression responses to the data from the current study, with gene expression at rest predominated by myogenic programs, and myogenic exercise responses being small in magnitude and characterized by inflammatory and stress related genes.

### 4.4 Limitations

First, we had a small sample of participants in the pre‐operative exercise cohort. This was further complicated by the data collection time‐frame being during the height of the COVID‐19 pandemic, in which strict precautions were implemented for non‐essential research, limiting recruitment. In particular, this may have influenced the power necessary to observe significant relationships between clinical phenotypes and gene profiles, and prohibited exploratory correlations between individual genes and clinical features. However, the observation of significant differences in gene expression for some of the gene programs may indicate sufficient power for our primary outcomes. As previously mentioned, the lack of a healthy control group precludes direct comparisons of exercise response in paraspinal muscle in individuals without pain or pathology. If, for example, the transcriptional response of these muscles is fundamentally different than more classically studied muscles (i.e., vastus lateralis, soleus, etc.), then our interpretation of the responses measured here would be incorrect. However, there is no experimental data to support or refute this possibility, so there is value in simply understanding the response of the muscle in any group of patients or controls. Secondarily, it is unknown whether the functional relevance of the observed gene expression changes is related to resulting downstream protein regulation, tissue adaptation, regeneration, or physiology. Additional mechanistic studies are needed to evaluate how these gene expression patterns translate to functional adaptive capacity.

## 5 CONCLUSION

Individuals undergoing surgery for LSP demonstrate low levels of overall gene expression at rest, but are dominated by myogenic and metabolic programs. In response to an acute bout of exercise, paraspinal muscle does not demonstrate a stereotypic myogenic response, and instead programs associated with lipid metabolism, inflammation, and extracellular matrix adaptation are induced. These data are valuable because they establish some degree of corroboration between exercise‐induced biological events, and the clinical observation that these individuals fail to recover muscle size and function. They are also valuable because they are the first gene expression data in these important muscles following exercise in patients with LSP. Future research is needed to explore the responsiveness of paraspinal muscles compared to more traditionally studied muscle (i.e., vastus lateralis, soleus, etc.) in healthy controls, and in patients with pathology. Similarly, further exploration of the upstream (i.e., signaling responses), downstream (protein synthesis), and physiological and functional consequences of these transcriptional responses to exercise, as well as the response mechanisms in the presence of musculoskeletal pain and pathology is needed.

## FUNDING STATEMENT

This project was funded by NIH R01HD088437.

## CONFLICT OF INTEREST STATEMENT

The authors declare no conflicts of interest.
